# Evolution of income inequalities in the caries experience of 5-year-old children in Brazil between 2010 and 2023

**DOI:** 10.1590/1980-549720260025.supl.1

**Published:** 2026-07-24

**Authors:** Armando Cabral de Lira, Isabela Ficagna Oshiro, Luiza De Carli Grieleitow, Maria Letícia Barbosa Raymundo, Amanda Barbosa Oliveira, Yuri Wanderley Cavalcanti, Roger Keller Celeste, Rafael Aiello Bomfim

**Affiliations:** I Universidade Federal da Paraíba, Department of Social Clinics and Dentistry - João Pessoa (PB), Brazil.; II Universidade Federal de Mato Grosso do Sul, Department of Preventive Dentistry - Campo Grande (MS), Brazil.; III Universidade Federal do Rio Grande do Sul, Department of Preventive and Social Dentistry - Porto Alegre (RS), Brazil.

**Keywords:** Dental caries, Oral health, Unified Health System, Public health policies

## Abstract

**Objective:**

To compare income inequalities in caries experience and untreated caries in 5-year-old children between the 2010 and 2023 SB Brasil surveys.

**Methods:**

A comparative analysis of the 2010 and 2023 SB Brasil surveys was performed. For each year, the mean decayed, extracted, and filled teeth (deft) index and its respective 95% confidence intervals (CI) were calculated, considering sampling weights and the complex design. Equivalent per capita family income was divided into four categories: below the poverty line; up to 1 minimum wage; up to 2 minimum wages; and above 2 minimum wages, to generate ridit scores according to sampling weights. The Slope Index of Inequality (SII) and the Relative Index of Inequality (RII) were estimated using linear regression and generalized linear models, respectively, for each year.

**Results:**

The mean deft decreased from 2.42 (95%CI 2.02-2.81) in 2010 to 2.14 (95%CI 1.92-2.36) in 2023. However, the SII remained stable, ranging from -1.32 (95%CI -1.70; -0.94) in 2010 to -1.14 (95%CI -1.55; -0.72) in 2023. Similarly, the RII also showed slight variation- 0.63 (95%CI 0.57-0.70) in 2010 and 0.61 (95%CI 0.51-0.73) in 2023-indicating that children from higher-income families consistently had about 40% lower risk of experiencing dental caries in both periods. The sensitivity analyses with untreated caries corroborated the findings.

**Conclusion:**

Despite the average reduction in caries experience, socioeconomic disparities remained unchanged between 2010 and 2023. These findings suggest that the implemented public policies were not sufficient to reduce income inequalities in children caries and the need for more innovative and equitable strategies.

## INTRODUCTION

Dental caries in early childhood remains one of the main public health issues in middle- and low-income countries, reflecting profound social inequalities and access to care^1^. In Brazil, despite the expansion of the Family Health Strategy and the implementation of policies aimed at promoting oral health, the prevalence of the disease remains high (deft index above 2) in childhood. This scenario is especially worrisome in vulnerable populations, particularly those without access to water fluoridation, where the burden of the disease is more pronounced and unequal^1^. Dental caries in early childhood compromises not only oral health, but also growth, cognitive development, school performance, and the quality of life of children^2^ ^,^ ^3^. Income is a relevant and widely used marker of social inequalities in oral health, with consistent evidence of association with worse oral outcomes^4^. However, it does not exhaust the complexity of the social position, and its findings should be interpreted as a specific dimension of socioeconomic inequalities^4^. Thus, understanding and addressing these inequalities through effective and equitable preventive strategies is a priority for Brazilian public health.

To explain these differences between social groups more accurately, indicators-such as the Slope Index of Inequality (SII), which quantifies absolute inequality, and the Relative Index of Inequality (RII), which expresses relative inequality-are increasingly used in oral health epidemiology^5^ ^,^ ^6^ ^,^ ^7^. These indicators have the advantage of measuring the magnitude of health inequalities with a single indicator, even when the socioeconomic variable is ordinal, avoiding unnecessary dichotomizations with traditional epidemiological methods. Researchers have shown that policies, such as water fluoridation and the expansion of dental services, had significant impacts, but are still insufficient to eliminate inequalities^8^ ^,^ ^9^.

In this context, it is worth analyzing the evolution of income inequalities in the experience of dental caries between 2010 and 2023, focusing on 5-year-old children. This age group is particularly sensitive to preventive interventions and quickly reflects the effects of public policies on oral health promotion and prevention, such as supervised tooth brushing in day care centers and nursery schools and access to fluoride, as well as ultra-processed food consumption patterns^10^ ^-^ ^11^. Assessing both absolute inequality (SII) and relative inequality (RII) allows us to verify not only whether the severity of the disease has decreased, but also whether it has decreased among the poorest and the richest.

In the present study, we aimed to compare income inequalities in the experience of caries and untreated caries in 5-year-old children between the 2010 and 2023 editions of the National Survey of Oral Health (SB Brasil), using the Slope (SII - absolute inequality) and Relative (RII - relative inequality) indices in the aforementioned surveys. Considering the expansion of Primary Health Care, oral health teams, the School Health Program, and income transfer policies in the period, we hypothesized that income inequalities in caries experience in 5-year-old children would have reduced between 2010 and 2023.

## METHODS

A comparative study was carried out with data from the National Survey of Oral Health (SB Brasil) 2010^12^ and 2023^13^, aiming at evaluating the evolution of income inequalities in caries experience in 5-year-old children in Brazil. The main outcome was the decayed, extracted, and filled teeth (deft) index, corresponding to the number of decayed, missing, and filled deciduous teeth due to caries. In addition, as the sensitivity outcome, the “d” component of the index was analyzed, referring to untreated caries. For each year, deft averages and their respective 95% confidence intervals (95%CI) were estimated, incorporating the sample weights and the complex design structure of each survey. It was not possible to include the surveys of 1986 and 2003 because, in 1986, the age group of 5 years was not included; and in 2003, there was no income information for this age group.

### Socioeconomic exposure variable

The exposure variable was the equivalent per capita family income. In order to increase the comparability between the surveys, the procedure for harmonizing income was adopted in both periods. In SB Brasil 2010, family income was originally collected in categories. In SB Brasil 2023, family income was made available in a continuous format, allowing the direct calculation of the equivalent per capita family income.

After harmonization, income was categorized into four strata distributed in an ordinal way: below the poverty line, up to 1 minimum wage, up to 2 minimum wages, and above 2 minimum wages. The minimum wage values in force for each period were considered, of BRL 510 in 2010 and BRL 1,320 in 2023. This categorization was adopted due to its considerable relevance in the Brazilian context, as it covers socially recognized milestones of economic vulnerability and social stratification^14^ ^,^ ^15^ ^,^ ^16^.

For analytical purposes, these categories were converted into continuous values from the average points of each range, and the equivalent per capita income was subsequently calculated, according to the methodological procedure described by Celeste and Bastos^17^.

Based on these ordered categories, the ridit score was calculated, weighted by the sample weights, seeking to express the relative position of each income stratum in the population distribution. This procedure allowed us to estimate inequality gradients throughout the entire socioeconomic hierarchy, avoiding comparisons restricted only to extreme groups.

### Statistical analysis

Descriptive analyses were presented through univariate and bivariate distributions, with estimates followed by 95%CI. For each year, absolute and relative inequalities in caries experience were estimated using the SII and RII indices^5^ ^,^ ^6^ ^,^ ^7^.

The indices were calculated with generalized linear models, considering the complex design of the sample and the incorporation of the sample weights. For the SII, a Poisson distribution model, robust variance, and identity link function were used, in such a way that the estimated coefficient represented the absolute mean difference in the number of affected teeth between the extremes of socioeconomic distribution. In turn, for the RII, a Poisson distribution model, robust variance, and logarithmic link function were used, in which the exponential coefficient expressed the relative ratio of means between the extremes of the income hierarchy. This strategy is appropriate for counting outcomes, such as the deft, and allows for consistent estimates of absolute and relative inequalities^17^.

Moreover, sensitivity analysis was carried out using the presence of untreated caries, defined according to the “d” component the deft index All analyses were performed using Stata 14.2 software (College Station, TX, USA). The analytical plan incorporated the complex sampling structure of the surveys, in order to produce representative estimates of the Brazilian population of 5-year-old children.

### Comparability between surveys

The comparison between the SB Brasil 2010 and 2023 surveys was conducted based on common methodological elements that support their comparability at the population level. Both surveys are national population-based surveys, with complex, probabilistic, and cluster sampling, conducting home oral examinations and interviews, and using similar diagnostic criteria for the main oral diseases and age groups analyzed. Authors of a recent study on the methodological aspects of the SB Brasil editions highlight that, despite differences between the surveys regarding the sampling plan, the study domains, the listing process, the composition of the field teams, and the incorporation of technologies for data recording and monitoring, the main indicators and conditions evaluated were maintained, which favors the temporal comparability of the results. Conversely, such operational and methodological differences recommend caution in the interpretation of comparative estimates between the two periods, especially in the face of possible influences of non-response, organization of fieldwork, and contextual changes that occurred between 2010 and 2023^13^. As the income categories were arranged from the worst to the best socioeconomic position, negative values of SII and RII values below 1 indicate that the dental caries experience is disproportionately concentrated in the lower-income groups.

## Data Availability Statement:

The entire dataset that supports the results of this study originates from the national surveys SB Brasil 2010 and SB Brasil 2023, conducted by the Ministry of Health. These are secondary and anonymized databases, available at their own institutional platforms.

## RESULTS

In Table 1, we show that the average of the deft index did not significantly vary between the two analyzed surveys. In 2010, the average value was 2.42 (95%CI 2.02-2.81), while in 2023 it was 2.14 (95%CI 1.92-2.36).

**Table 1.** Average and prevalence of the deft index in 5-year-old children in the SB Brasil 2010 and 2023 surveys.

Year	Average deft	95%CI	Prevalence (%)	95%CI
2010	2.42	2.02-2.81	53.1	47.5-58.6
2023	2.14	1.92-2.36	46.80	43.1-50.6

deft: decayed, extracted, and filled teeth; 95%CI: 95% confidence interval.

In Table 2, we demonstrate that, when stratifying the caries experience according to per capita family income, a clear socioeconomic gradient was observed both in 2010 and in 2023. In 2010, children below the poverty line presented the highest average deft value (3.16; 95%CI 2.32-4.00), followed by those of families with income up to 1 minimum wage (2.77; 95%CI 2.51-3.03). Among children belonging to the categories of 1 to 2 minimum wages and above 2 minimum wages, the values were substantially lower, corresponding to 1.85 (95%CI 1.51-2.19) and 1.13 (95%CI 0.86-1.40), respectively. In 2023, we observed the same pattern of inequality: children below the poverty line presented average deft of 2.86 (95%CI 2.41-3.30), contrasting with 1.18 (95%CI 0.79-1.57) among those belonging to families with incomes above 2 minimum wages

**Table 2.** Average of the deft index in 5-year-old children according to per capita family income ranges. Brazil, SB Brasil 2010 and 2023.

Year	2010			2023		
Income range	n	deft	95%CI	n	deft	95%CI
Below the poverty line	1,487	3.16	2.32-4.00	1,526	2.86	2.41-3.30
Poverty line up to 1 MW	2,993	2.77	2.51-3.03	1,520	2.28	1.81-2.75
From 1 to 2 MWs	1,646	1.85	1.51-2.19	1,140	1.82	1.44-2.19
Above 2 MWs	876	1.13	0.86-1.40	608	1.18	0.79-1.57

deft: decayed, extracted, and filled teeth; MW: minimum wage; 95%CI: 95% confidence interval.

The SII was -1.32 (95%CI -1.70; -0.94) in 2010 and -1.14 (95%CI -1.55; -0.72) in 2023, with a non-significant reduction in the absolute difference in caries experience among socioeconomic strata. Similarly, the RII remained stable, ranging from 0.63 (95%CI 0.57-0.70) in 2010 to 0.61 (95%CI 0.51-0.73) in 2023. These results indicate that there was no change in the inequality overview. Furthermore, children from families with higher income continued to show an approximately 40% lower odds of experiencing the disease, configuring a persistent pattern of inequality. As per the sensitivity analysis (Table 3), considering untreated caries, we found similar results.

**Table 3.** Indices of absolute inequality (Slope Index of Inequality) and relative inequality (Relative Index of Inequality) in the experience of caries and untreated caries in 5-year-old children, SB Brasil 2010 and 2023.

Outcome	Year	SII (95%CI)	RII (95%CI)
Caries experience	2010	-1.32 (-1.70; -0.94)	0.63 (0.57-0.70)
Caries experience	2023	-1.14 (-1.55; -0.72)	0.61 (0.51-0.73)
Untreated caries	2010	-1.38 (-1.77; -0.99)	0.58 (0.52-0.63)
Untreated caries	2023	-1.18 (-1.50; -0.86)	0.51 (0.42-0.62)

SII [Slope Index of Inequality]: represents the absolute difference in caries experience between income extremes; RII [Relative Index of Inequality]: expresses the relative risk ratio of caries among children of families with lower and higher income; 95%CI: 95% confidence interval.

## DISCUSSION

According to the data analysis, between 2010 and 2023, there was no significant reduction in the average of the deft index in 5-year-old children. Taking this result into consideration, the potential advances in the Brazilian oral health policy did not result in effects on the control of dental caries in the Brazilian child population. Within this context, the data of this study evidence that the expansion of oral health coverage in Primary Health Care^18^ and the implementation of the School Health Program^19^ did not produce effects on caries experience data in 5-year-old children in Brazil. Besides, inequalities marked by income also remained stable during the analyzed period.

The study has strengths and limitations. First, structural changes of socioeconomic nature, such as changes in social protection policies, variations in poverty and income levels, changes in access to Primary Health Care and oral health, as well as changes in children’s nutrition pattern, tend to produce gradual effects, which may not be fully contemplated at relatively short time intervals between national surveys. Moreover, there is still a shortage of standardized and widely available indicators that allow the continuous monitoring of structural changes related to social determinants of oral health at the population level. Second, the 2023 survey showed a higher non-response rate, a phenomenon observed in contemporary epidemiological surveys in different countries. Although the non-response may introduce selection bias, the complex sampling design and the application of sample weights contribute to preserving the population representativeness of the estimates. Lastly, family income was used as a socioeconomic indicator; however, it is recognized that this variable alone may not comprehensively cover the multiple dimensions of the social conditions of the evaluated children. Nevertheless, in 2010 it was not possible to consider maternal level of education, an indicator commonly adopted in studies on this age group, which limited the comparability between the analyzed periods.

In addition, the available data are restricted to information obtained from population surveys and, therefore, do not allow contemplating contextual or collective-level determinants (such as characteristics of territories, access to social policies, or racial inequalities), which could deepen the understanding of the observed disparities.

Among the strengths, it is a comparative analysis of two national surveys conducted with representative samples of the population, which confers robustness and relevance on the presented estimates. Second, the use of standardized indicators of caries experience allows direct comparability between the years 2010 and 2023, strengthening the temporal interpretation of inequalities. Third, the application of absolute inequality (SII) and relative inequality (RII) measures, adjusted for the variability in the number of deciduous teeth, ensures greater accuracy and credibility in the measurement of social disparities in oral health. Fourth, the results contribute to filling a gap in national and international literature on the evolution of oral health inequalities in childhood, providing useful evidence to subsidize public health equity policies.

Furthermore, the comparative interpretation between SB Brasil 2010 and 2023 surveys should be done with caution^13^. Although both share key methodological characteristics, such as complex probabilistic cluster sampling, national coverage, home oral examinations and interviews, as well as maintenance of similar clinical indicators for the evaluation of dental caries, the editions were not operationally identical. As described by Vargas et al.^13^, there were changes in the sampling plan, the study domains, the household listing process, the composition of the field teams, and in the use of technologies for data collection and monitoring, especially in 2023. Moreover, possible contextual changes that occurred in the interval between surveys, including socioeconomic-related, changes in the organization of Primary Health Care, and in the child food environment, may also have influenced the distribution of caries and the observed inequalities.

The findings of this study should be interpreted considering that income is a relevant marker of social inequalities in oral health, with a consistent association with worse oral outcomes, but that does not exhaust the complexity of social position^4^. Hence, the inequalities observed between income strata represent an important dimension of socioeconomic stratification, without fully covering other structural and contextual factors that also influence the occurrence of caries in early childhood. This finding indicates that the benefits of actions and policies aimed at reducing caries have not equitably achieved the different social groups, with income remaining a key determinant of the protection against caries experience in Latin American countries^20^ ^,^ ^21^ ^,^ ^22^ and others^23^ ^,^ ^24^. In 2010, children from families with higher income had about 69% less chance of developing caries, a proportion that remained practically stable in 2023, with 66%.

This scenario points to the urgent need to improve the work process in oral health care of children, especially regarding prevention activities aimed at children in situations of socioeconomic vulnerability. In Brazil, however, there are still important barriers, such as the absence of supervised tooth brushing in many public schools, which represents a strategic gap in care and could be overcome with the expansion and qualification of preventive programs aimed at early childhood such as the School Health Program.

In addition, cultural aspects are relevant and often underestimate the importance of deciduous teeth, which is often seen as transient teeth and of lower clinical value for caregivers and professionals, a perception that can harm preventive strategies. Nonetheless, this perception ignores the fact that the presence of caries in deciduous teeth is a strong predictor of the future occurrence of caries in permanent teeth^25^. Therefore, adequate care to oral health in early childhood should be understood not only as immediate prevention, but also as an investment to reduce lifelong illnesses.

Commercial health determinants play a key role in the persistence and worsening of dental caries. The availability, accessibility, and intense advertising of ultra-processed products rich in added sugars are one of the main drivers of the global caries epidemic^26^. According to recent findings with data from SB Brasil 2023, nonwhite children, those who are beneficiaries of social programs, and with limited use of dental services are more vulnerable to dental caries, its clinical consequences, and the need for immediate treatment. This pattern reinforces the need for equity-oriented public policies, with increased access to prevention and timely dental care, especially among more socially-vulnerable groups^27^.

In addition to noticing the absence of significant reduction in the deft averages between the years 2010 and 2023, we observed that inequities associated with income remained unchanged in the period. This persistence evidences that, although public policies-such as the expansion of Primary Health Care and income transfer programs^28^-have contributed to social advances, they were not enough to eliminate the structural barriers that affect the risk of caries in the most vulnerable groups.

The maintenance of lower values among children of higher-income families, without significant reduction of disparities over the period, points to the need for more focused and equitable policies, combining universal prevention strategies, such as water fluoridation and supervised tooth brushing school programs, proportional actions aimed at contexts of greater vulnerability. In this sense, we highlight the recent review conducted by Couto et al.^29^, who synthesized evidence about fluoride interventions in preschool children and reinforced that the regular use of fluoride toothpaste remains the central preventive measure, with additional strategies, such as fluoride varnish, being complementary and non-substitutive. Supervised tooth brushing enhances the effectiveness of fluoride toothpaste by ensuring adequate frequency, correct amount of toothpaste, and appropriate technique, in addition to reducing inequalities in fluoride access in socially-vulnerable contexts^29^.

Researchers analyzed the effect of the coverage of income transfer programs on the provision of dental procedures from the perspective of the Brazilian Unified Health System (SUS)^28^. The authors concluded that municipalities that expanded the coverage of income transfer programs, which aim to reduce inequalities, contributed to the increase of dental restorations and extractions, without having a significant effect on the provision of individual or collective preventive procedures. Minimum wage valorization policies, including inflation corrections and actual increases, have direct influence on the purchasing power of households with lower income, significantly changing the per capita income. These public policies create natural parameters to categorize family income, reflecting recognized socioeconomic milestones and objectives, which were used in the present study^14^ ^,^ ^15^. It should be noted that between 2010 and 2023, programs-such as Bolsa Família (income transfer program of the Brazilian government)-played a key role in expanding the transfer of income to vulnerable families, contributing to social advancement and poverty mitigation^16^.

 The literature is still limited and presents heterogeneous evidence on the effect of income transfer programs on child oral health^30^. Although the present study

was not designed to directly assess the impact of these policies, our findings indicate that income inequalities remain associated with the occurrence of

dental caries in early childhood. According to available evidence, income transfer programs may contribute to reducing the occurrence of untreated caries in families in extreme poverty^31^; however, such initiatives alone are not sufficient to eliminate the structural barriers that support the socioeconomic gradient of the disease, reinforcing the need for intersectoral actions articulated with the organization of oral health services. Therefore, more effective measures to reduce inequalities should be implemented concomitantly with more effective prevention strategies. Furthermore, future studies may observe the regional variation of these inequalities, comparing different regions of the country.

All in all, socioeconomic inequalities in oral health remained unchanged between 2010 and 2023. These findings suggest that the implemented public policies were not sufficient, emphasizing the need for more innovative and equitable strategies.

## References

[1] Narvai PC, Frazão P, Roncalli AG, Antunes JL (2006). Cárie dentária no Brasil: declínio, polarização, iniqüidade e exclusão social [Dental caries in Brazil: decline, polarization, inequality and social exclusion]. Rev Panam Salud Publica.

[2] Piva F, Pereira JT, Luz PB, Hashizume LN, Hugo FN, Araujo FB (2017). A longitudinal study of early childhood caries and associated factors in Brazilian children. Braz Dent J.

[3] Karam SA, Costa FS, Chisini LA, Darley R, Demarco FF, Correa MB (2024). Can oral health have an impact on academic performance and school absenteeism? A systematic review and meta-analysis. Braz J Oral Sci.

[4] Singh A, Peres MA, Watt RG (2019). The relationship between income and oral health: a critical review. J Dent Res.

[5] Blair YI, McMahon AD, Macpherson LM (2013). Comparison and relative utility of inequality measurements: as applied to Scotland’s child dental health. PLoS One.

[6] Keppel KG, Pamuk E, Lynch J, Carter-Pokras O, Kim I, Mays V (2005). Methodological issues in measuring health disparities. Vital Health Stat 2.

[7] Silva ICM, Restrepo-Mendez MC, Costa JC, Ewerling F, Hellwig F, Ferreira LZ (2018). Mensuração de desigualdades sociais em saúde: conceitos e abordagens metodológicas no contexto brasileiro. Epidemiol Serv Saúde.

[8] Bomfim RA, Watt RG, Tsakos G, Heilmann A, Frazão P (2022). Does water fluoridation influence ethnic inequalities in caries in Brazilian children and adolescents?. Community Dent Oral Epidemiol.

[9] Bomfim RA, Frazão P (2022). Impact of water fluoridation on dental caries decline across racial and income subgroups of Brazilian adolescents. Epidemiol Health.

[10] Cascaes AM, Silva NRJD, Fernandez MDS, Bomfim RA, Vaz JDS (2022). Ultra-processed food consumption and dental caries in children and adolescents: a systematic review and meta-analysis. Br J Nutr.

[11] de Souza MS, Vaz JDS, Martins-Silva T, Bomfim RA, Cascaes AM (2021). Ultra-processed foods and early childhood caries in 0-3-year-olds enrolled at Primary Healthcare Centers in Southern Brazil. Public Health Nutr.

[12] Roncalli AG, Silva NN, Nascimento AC, Freitas CH, Casotti E, Peres KG (2012). Aspectos metodológicos do Projeto SBBrasil 2010 de interesse para inquéritos nacionais de saúde [Relevant methodological issues from the SBBrasil 2010 Project for national health surveys]. Cad Saúde Pública.

[13] Vargas AMD, Teixeira DSDC, Alves MCGP, Alencar GP, Bernal RTI, Vasconcelos M (2025). Methodological aspects of national surveys in Brazil: contributions to the debate on oral health surveillance. Braz Oral Res.

[14] Instituto de Pesquisa Econômica Aplicada (IPEA) (2024). Renda, Pobreza e Desigualdade - Indicadores.

[15] Ministério da Cidadania (2023). Programa Bolsa Família - Impactos na redução da pobreza.

[16] Costa DM, Magalhães R, Cardoso MLM (2023). From the Brazilian Income Transfer Program to Brazil Assistance: challenges and achievements according to a theory-driven evaluation research on the program. Cad Saúde Pública.

[17] Celeste RK, Bastos JL (2013). Mid-point for open-ended income category and the effect of equivalence scales on the income-health relationship. Rev Saude Publica.

[18] Menezes LXB, Corrêa GT, Lucena EHG, Celeste RK, Cavalcanti YW (2025). Tendência temporal de equipes de saúde bucal da Estratégia Saúde da Família nos municípios brasileiros de 2001 a 2021 [Time trend of Family Health Strategy oral health teams in Brazilian municipalities from 2001 to 2021]. Cad Saude Publica.

[19] Lattanzi AP, Marques APF, Silveira FM, Valente MIB, Antunes LA, Cortellazzi KL (2020). The influence of the Brazilian school health program on the oral-health-related quality of life of adolescents. Braz Oral Res.

[20] Martignon S, Guarnizo-Herreño CC, Franco-Cortés AM, García-Zapata LM, Ochoa-Acosta EM, Restrepo-Pérez LF (2024). Socioeconomic inequalities in early childhood caries: evidence from vulnerable populations in Colombia. Braz Oral Res.

[21] Aravena-Rivas Y, Monsalves MJ, Espinoza-Espinoza G, Weitz A, Hernández B, Castillo J (2022). Impact of socioeconomic inequalities on dental caries in deprived children: a multilevel analysis. Community Dent Health.

[22] Martignon S, Roncalli AG, Alvarez E, Aránguiz V, Feldens CA, Buzalaf MAR (2021). Risk factors for dental caries in Latin American and Caribbean countries. Braz Oral Res.

[23] Chang Q, Cheng M, Xu M, Du S, Wang X, Feng X (2023). Decomposing socioeconomic inequalities in dental caries among Chinese adults: findings from the 4th national oral health survey. BMC Oral Health.

[24] Peres MA, Ju X, Mittinty M, Spencer AJ, Do LG (2019). Modifiable Factors Explain Socioeconomic Inequalities in Children’s Dental Caries. J Dent Res.

[25] Hall-Scullin E, Whitehead H, Milsom K, Tickle M, Su TL, Walsh T (2017). Longitudinal Study of Caries Development from Childhood to Adolescence. J Dent Res.

[26] Moynihan PJ, Kelly SA (2014). Effect on caries of restricting sugars intake: systematic review to inform WHO guidelines. J Dent Res.

[27] Moura RNV, Paiva SM, Ramos-Jorge J, Pinto RDS, Lara JVI, Barbosa MCF (2025). Social inequities and dental caries in 5-year-old children: a study with results from SB Brasil 2023. Braz Oral Res.

[28] Colvara BC, Ritzel IF, Aguiar VR, Hilgert JB, Celeste RK (2023). Cobertura do Programa Bolsa Família e fatores associados à realização de procedimentos odontológicos no Brasil, entre 2007 e 2011: um estudo ecológico [Coverage of the Brazilian Income Transfer Program and factors associated with the performance of dental procedures in Brazil, from 2007 to 2011: an ecological study]. Cad Saude Publica.

[29] Couto FM, Sousa FSO, Dhyppolito IM, Barja-Fidalgo F, Dos Santos APP, Nadanovsky P (2026). Fluoride Varnish for Caries Prevention in Preschoolers: An Overview of Reviews. Community Dent Oral Epidemiol.

[30] Colvara BC, Singh A, Gupta A, Celeste RK, Hilgert JB (2023). Association between cash transfer programs and oral health-A scoping review. J Public Health Dent.

[31] Calvasina P, O’Campo P, Pontes MM, Oliveira JB, Vieira-Meyer APGF (2018). The association of the Bolsa Familia Program with children’s oral health in Brazil. BMC Public Health.

