# Supplementary material for: Evolution of income inequalities in the caries experience of 5-year-old children in Brazil between 2010 and 2023
Source: Rev Bras Epidemiol. 2026 Jul 24;29(Suppl 1):e260025supl1. doi: 10.1590/1980-549720260025.supl.1 (PMC13403699; doi:10.1590/1980-549720260025.supl.1)
Supplement: Translation

# Translation: Evolução das desigualdades de renda na experiência de cárie em crianças de 5 anos no Brasil entre 2010 e 2023

**DOI:** 10.1590/1980-549720260025.supl.1.2

**Authors:** Armando Cabral de Lira, Isabela Ficagna Oshiro, Luiza De Carli Grieleitow, Maria Letícia Barbosa Raymundo, Amanda Barbosa Oliveira, Yuri Wanderley Cavalcanti, Roger Keller Celeste, Rafael Aiello Bomfim

**Affiliations:** I Universidade Federal da Paraíba, Departamento de Clínica e Odontologia Social - João Pessoa (PB), Brasil.; II Universidade Federal de Mato Grosso do Sul, Departamento de Odontologia Preventiva - Campo Grande (MS), Brasil.; III Universidade Federal do Rio Grande do Sul, Departamento de Odontologia Preventiva e Social - Porto Alegre (RS), Brasil.

**Keywords:** Cárie dentária, Saúde bucal, Sistema Único de Saúde, Políticas públicas de saúde

## Abstract

**Objetivo:**

Comparar as desigualdades de renda na experiência de cárie e cárie não tratada em crianças de 5 anos entre os inquéritos da Pesquisa Nacional de Saúde Bucal (SB Brasil) 2010 e 2023.

**Métodos:**

Foi realizada uma análise comparativa dos inquéritos SB Brasil 2010 e 2023. Calculou-se a média do índice ceo-d e seus respectivos Intervalos de Confiança de 95% (IC95%), considerando pesos amostrais e delineamento complexo. A renda familiar per capita equivalente foi categorizada em quatro faixas: abaixo da linha da pobreza, até 1 salário mínimo, até 2 salários mínimos e acima de 2 salários mínimos, permitindo a construção de escores ridit ponderados. O Slope Index of Inequality (SII) e o Relative Index of Inequality (RII) foram estimados por Modelos Lineares Generalizados (GLM) com distribuição Poisson e variância robusta, separadamente para cada ano.

**Resultados:**

O ceo-d médio não apresentou variação, passando de 2,42 (IC95% 2,02-2,81) em 2010 para 2,14 (IC95% 1,92-2,36) em 2023. O índice SII permaneceu relativamente estável, variando de -1,32 (IC95% -1,70; -0,94) em 2010 para -1,14 (IC95% -1,55; -0,72) em 2023. Da mesma forma, o RII mostrou pouca variação: 0,63 (IC95% 0,57-0,70) em 2010 e 0,61 (IC95% 0,51-0,73) em 2023, indicando que crianças com maior renda tiveram cerca de 40% menor risco de desenvolver cárie em ambos os períodos. Análises de sensibilidade com cárie não tratada confirmaram os achados.

**Conclusão:**

As desigualdades socioeconômicas em saúde bucal mantiveram-se inalteradas entre 2010 e 2023. Esses resultados sugerem que políticas públicas implementadas no período podem não ter sido suficientes, reforçando a necessidade de estratégias inovadoras e mais equitativas.

## INTRODUÇÃO

A cárie dentária na primeira infância continua sendo um dos principais problemas de saúde pública em países de média e baixa renda, refletindo profundas desigualdades sociais e de acesso ao cuidado^1^. No Brasil, apesar da expansão da Estratégia Saúde da Família e da implementação de políticas voltadas à promoção da saúde bucal, a prevalência da doença permanece elevada (ceo-d acima de 2) na infância. Esse cenário é especialmente preocupante em populações vulneráveis, particularmente naquelas sem acesso à água fluoretada, onde a carga da doença é mais intensa e desigual^1^. A cárie dentária na primeira infância compromete não apenas a saúde bucal, mas também o crescimento, o desenvolvimento cognitivo, o desempenho escolar e a qualidade de vida das crianças^2^ ^,^ ^3^. A renda constitui um marcador relevante e amplamente utilizado das desigualdades sociais em saúde bucal, com evidência consistente de associação com piores desfechos bucais^4^. No entanto, ela não esgota a complexidade da posição social, devendo seus achados ser interpretados como uma dimensão específica das desigualdades socioeconômicas^4^. Assim, compreender e enfrentar essas desigualdades por meio de estratégias preventivas eficazes e equitativas constitui uma prioridade para a saúde pública brasileira.

Para captar essas diferenças entre grupos sociais de forma mais precisa, indicadores como o Slope Index of Inequality (SII), que quantifica a desigualdade absoluta, e o Relative Index of Inequality (RII), que expressa a desigualdade relativa, são cada vez mais usados em epidemiologia da saúde bucal^5^ ^,^ ^6^ ^,^ ^7^. Esses indicadores possuem a vantagem de medir a magnitude das desigualdades em saúde com um indicador único, mesmo quando a variável socioeconômica é ordinal, evitando dicotomizações desnecessárias com métodos epidemiológicos tradicionais. Pesquisas demonstram que políticas como a fluoretação da água e a expansão dos serviços odontológicos tiveram impactos relevantes, mas ainda insuficientes para eliminar as desigualdades^8^ ^,^ ^9^.

Nesse contexto, torna-se relevante analisar a evolução das desigualdades de renda na experiência de cárie entre 2010 e 2023, focalizando crianças de 5 anos. Essa faixa etária é particularmente sensível para intervenções preventivas e reflete rapidamente os efeitos das políticas públicas de promoção e prevenção em saúde bucal, como a escovação supervisionada em creches e pré-escolas e acesso a fluoretos, bem como padrão de consumo de alimentos ultraprocessados^10^ ^-^ ^11^. Avaliar tanto a desigualdade absoluta (SII) quanto a relativa (RII) permite verificar não apenas se a gravidade da doença diminuiu, mas se ela diminuiu igualmente entre os mais pobres e os mais ricos.

O objetivo do presente estudo foi comparar as desigualdades de renda na experiência de cárie e cárie não tratada em crianças de 5 anos entre os inquéritos da Pesquisa Nacional de Saúde Bucal (SB Brasil) 2010 e 2023, utilizando os índices Slope (SII - desigualdade absoluta) e Relative (RII - desigualdade relativa) nos referidos inquéritos. Considerando a expansão da atenção primária, das equipes de saúde bucal, do Programa Saúde na Escola e das políticas de transferência de renda no período, partimos da hipótese de que as desigualdades de renda na experiência de cárie em crianças de 5 anos teriam reduzido entre 2010 e 2023.

## MÉTODOS

Foi realizado um estudo comparativo com dados dos inquéritos nacionais de saúde bucal SB Brasil 2010^12^ e SB Brasil 2023^13^, com o objetivo de avaliar a evolução das desigualdades de renda na experiência de cárie em crianças de 5 anos no Brasil. O desfecho principal foi o índice ceo-d, correspondente ao número de dentes decíduos cariados, extraídos por cárie ou restaurados. Também foi analisado, como desfecho de sensibilidade, o componente c do índice, referente à cárie não tratada. Para cada ano, foram estimadas as médias do ceo-d e seus respectivos Intervalos de Confiança de 95% (IC95%), incorporando os pesos amostrais e a estrutura de delineamento complexo de cada levantamento. Não foi possível incluir os levantamentos de 1986 e 2003, pois, em 1986, a faixa etária de 5 anos não foi contemplada, e em 2003 não houve coleta de informações sobre renda para esse grupo etário.

### Variável de exposição socioeconômica

A variável de exposição foi a renda familiar per capita equivalente. Para aumentar a comparabilidade entre os inquéritos, adotou-se procedimento de harmonização da renda nos dois períodos. No SB Brasil 2010, a renda familiar foi originalmente coletada em categorias. No SB Brasil 2023, a renda familiar foi disponibilizada em formato contínuo, permitindo o cálculo direto da renda familiar per capita equivalente.

Após essa harmonização, a renda foi categorizada em quatro estratos ordinalmente distribuídos: abaixo da linha da pobreza, até 1 salário mínimo, até 2 salários mínimos e acima de 2 salários mínimos. Foram considerados os valores do salário mínimo vigentes em cada período, de R$ 510 em 2010 e R$ 1.320 em 2023. Essa categorização foi adotada por sua relevância substantiva no contexto brasileiro, uma vez que contempla marcos socialmente reconhecidos de vulnerabilidade econômica e estratificação social^14^ ^,^ ^15^ ^,^ ^16^.

Para fins analíticos, essas categorias foram convertidas em valores contínuos a partir dos pontos médios de cada faixa, sendo posteriormente calculada a renda per capita equivalente, conforme procedimento metodológico descrito por Celeste e Bastos^17^.

Com base nessas categorias ordenadas, foi calculado o escore ridit, ponderado pelos pesos amostrais, de modo a expressar a posição relativa de cada estrato de renda na distribuição populacional. Esse procedimento permitiu estimar gradientes de desigualdade ao longo de toda a hierarquia socioeconômica, evitando comparações restritas apenas aos grupos extremos.

### Análise estatística

As análises descritivas foram apresentadas por meio de distribuições univariadas e bivariadas, com estimativas acompanhadas de IC95%. Para cada ano, as desigualdades absolutas e relativas na experiência de cárie foram estimadas por meio dos índices SII e RII^5^ ^,^ ^6^ ^,^ ^7^.

O cálculo dos índices foi realizado com modelos lineares generalizados, considerando o delineamento complexo da amostra e a incorporação dos pesos amostrais. Para o SII, utilizou-se modelo com distribuição Poisson, variância robusta e função de ligação identidade, de modo que o coeficiente estimado representasse a diferença absoluta média no número de dentes afetados entre os extremos da distribuição socioeconômica. Já para o RII, utilizou-se modelo com distribuição Poisson, variância robusta e função de ligação logarítmica, em que o coeficiente exponenciado expressou a razão relativa de médias entre os extremos da hierarquia de renda. Essa estratégia é apropriada para desfechos de contagem, como o ceo-d, e permite estimar de forma consistente desigualdades absolutas e relativas^17^.

Adicionalmente, foi realizada análise de sensibilidade utilizando a presença de cárie não tratada, definida de acordo com o componente c do índice ceo-d. Todas as análises foram realizadas no software Stata 14.2 (College Station, TX, EUA). O plano analítico incorporou a estrutura de amostragem complexa dos inquéritos, de forma a produzir estimativas representativas da população brasileira de crianças de 5 anos.

### Comparabilidade entre os inquéritos

A comparação entre os inquéritos SB Brasil 2010 e 2023 foi conduzida com base em elementos metodológicos comuns que sustentam sua comparabilidade em nível populacional. Ambos os levantamentos são inquéritos nacionais de base populacional, com amostragem complexa, probabilística e por conglomerados, realização de exames bucais domiciliares e entrevistas, além da utilização de critérios diagnósticos semelhantes para os principais agravos bucais e faixas etárias analisadas. Estudo recente sobre os aspectos metodológicos das edições do SB Brasil destaca que, apesar das diferenças entre os levantamentos quanto ao plano amostral, aos domínios de estudo, ao processo de listagem, à composição das equipes de campo e à incorporação de tecnologias para registro e monitoramento dos dados, houve manutenção dos principais indicadores e condições avaliados, o que favorece a comparabilidade temporal dos resultados. Por outro lado, tais diferenças operacionais e metodológicas recomendam cautela na interpretação das estimativas comparativas entre os dois períodos, especialmente diante de possíveis influências da não resposta, da organização do trabalho de campo e de mudanças contextuais ocorridas entre 2010 e 2023^13^. Como as categorias de renda foram ordenadas da pior para a melhor posição socioeconômica, valores negativos de SII e valores de RII inferiores a 1 indicam que a experiência de cárie se concentra desproporcionalmente nos grupos de menor renda.

## Declaração de Disponibilidade de Dados:

Todo o conjunto de dados que dá suporte aos resultados deste estudo são provenientes dos inquéritos nacionais SB Brasil 2010 e SB Brasil 2023, conduzidos pelo Ministério da Saúde. Tratam-se de bases de dados secundárias e anonimizadas, disponibilizadas em plataformas institucionais próprias.

## RESULTADOS

A Tabela 1 mostra que a média do índice ceo-d não variou significativamente entre os dois inquéritos analisados. Em 2010, o valor médio foi de 2,42 (IC95% 2,02-2,81), enquanto em 2023 foi 2,14 (IC95% 1,92-2,36).

**Tabela 1.** Média e prevalência do índice ceo-d em crianças de 5 anos nos inquéritos SB Brasil 2010 e 2023.

Ano	ceo-d médio	IC95%	Prevalência (%)	IC95%
2010	2,42	2,02-2,81	53,1	47,5-58,6
2023	2,14	1,92-2,36	46,80	43,1-50,6

IC95%: intervalo de confiança de 95%.

A Tabela 2 mostrou que, ao estratificar a experiência de cárie segundo a renda familiar per capita, observou-se um nítido gradiente socioeconômico tanto em 2010 quanto em 2023. Em 2010, as crianças abaixo da linha da pobreza apresentaram o maior valor médio de ceo-d (3,16; IC95% 2,32-4,00), seguido por aquelas de famílias com renda até 1 salário mínimo (2,77; IC95% 2,51-3,03). Entre as crianças pertencentes às categorias de 1 a 2 salários mínimos e acima de 2 salários mínimos, os valores foram substancialmente menores, correspondendo a 1,85 (IC95% 1,51-2,19) e 1,13 (IC95% 0,86-1,40), respectivamente. Em 2023, manteve-se o mesmo padrão de desigualdade: crianças abaixo da linha da pobreza apresentaram ceo-d médio de 2,86 (IC95% 2,41-3,30), contrastando com 1,18 (IC95% 0,79-1,57) entre aquelas pertencentes às famílias com renda acima de 2 salários mínimos

**Tabela 2.** Média do índice ceo-d em crianças de 5 anos segundo faixas de renda familiar per capita. Brasil, SB Brasil 2010 e 2023.

Ano	2010			2023		
Faixa de renda	n	ceo-d	IC95%	n	ceo-d	IC95%
Abaixo da linha da pobreza	1.487	3,16	2,32-4,00	1.526	2,86	2,41-3,30
Linha da pobreza até 1 SM	2.993	2,77	2,51-3,03	1.520	2,28	1,81-2,75
De 1 a 2 SM	1.646	1,85	1,51-2,19	1.140	1,82	1,44-2,19
Acima de 2 SM	876	1,13	0,86-1,40	608	1,18	0,79-1,57

SM: salário mínimo; IC95%: intervalo de confiança de 95%.

O índice de desigualdade absoluta (SII) foi de -1,32 (IC95%: -1,70; -0,94) em 2010 e de -1,14 (IC95% -1,55; -0,72) em 2023, com redução não significativa na diferença absoluta da experiência de cárie entre os estratos socioeconômicos. De forma semelhante, o índice de desigualdade relativa (RII) permaneceu estável, passando de 0,63 (IC95% 0,57-0,70) em 2010 para 0,61 (IC95% 0,51-0,73) em 2023. Esses resultados indicam que não houve mudança no panorama da desigualdade. Ainda, crianças de famílias com maior renda continuaram apresentando uma probabilidade aproximadamente 40% menor de vivenciar a doença, configurando um padrão persistente de desigualdade. A análise de sensibilidade (Tabela 3), considerando a cárie não tratada, mostrou resultados semelhantes.

**Tabela 3.** Índices de desigualdade absoluta (SII) e relativa (RII) na experiência de cárie e cárie não tratada em crianças de 5 anos, SB Brasil 2010 e 2023.

Desfecho	Ano	SII (IC95%)	RII (IC95%)
Experiência de cárie	2010	-1,32 (-1,70; -0,94)	0,63 (0,57-0,70)
Experiência de cárie	2023	-1,14 (-1,55; -0,72)	0,61 (0,51-0,73)
Cárie não tratada	2010	-1,38 (-1,77; -0,99)	0,58 (0,52-0,63)
Cárie não tratada	2023	-1,18 (-1,50; -0,86)	0,51 (0,42-0,62)

SII: representa a diferença absoluta na experiência de cárie entre os extremos de renda; RII: expressa a razão relativa do risco de cárie entre crianças de famílias com menor e maior renda; IC95%: intervalo de confiança de 95%.

## DISCUSSÃO

A análise dos dados evidencia que, entre 2010 e 2023, não houve redução significativa na média do índice ceo-d em crianças de 5 anos. Esse resultado sugere que os potenciais avanços na política de saúde bucal brasileira não resultaram em efeitos no controle da cárie dentária na população infantil brasileira. Sob esse contexto, os dados deste estudo revelam que a ampliação da cobertura de saúde bucal na atenção primária^18^ e a implementação do Programa Saúde na Escola^19^ não produziram efeitos sobre os dados de experiência de cárie em crianças de 5 anos no Brasil. Além disso, as desigualdades marcadas pela renda também se mantiveram estáveis durante o período analisado.

O estudo apresenta forças e limitações. Primeiramente, mudanças estruturais de natureza socioeconômica, como transformações nas políticas de proteção social, variações nos níveis de pobreza e renda, mudanças no acesso à Atenção Primária à Saúde e à saúde bucal, bem como alterações no padrão alimentar infantil, tendem a produzir efeitos graduais, que podem não ser plenamente captados em intervalos temporais relativamente curtos entre inquéritos nacionais. Além disso, ainda há escassez de indicadores padronizados e amplamente disponíveis que permitam monitorar, de forma contínua, as mudanças estruturais relacionadas aos determinantes sociais da saúde bucal em nível populacional. Em segundo lugar, o inquérito de 2023 apresentou taxa de não resposta mais elevada, fenômeno observado em inquéritos epidemiológicos contemporâneos em diferentes países. Embora a não resposta possa introduzir viés de seleção, o desenho amostral complexo e a aplicação de ponderações amostrais contribuem para preservar a representatividade populacional das estimativas. Por fim, a renda familiar foi utilizada como indicador socioeconômico; contudo, reconhece-se que essa variável, isoladamente, pode não captar de forma abrangente as múltiplas dimensões das condições sociais das crianças avaliadas. Apesar disso, em 2010 não foi possível utilizar a escolaridade materna, indicador comumente adotado em estudos nessa faixa etária, o que limitou a comparabilidade entre os períodos analisados.

Outro ponto: os dados disponíveis se restringem a informações obtidas em inquéritos populacionais e, portanto, não permitem capturar determinantes contextuais ou de nível coletivo (como características dos territórios, acesso a políticas sociais ou desigualdades raciais), que poderiam aprofundar a compreensão das disparidades observadas.

Entre as forças, trata-se de uma análise comparativa de dois inquéritos nacionais conduzidos com amostras representativas da população, o que confere robustez e relevância às estimativas apresentadas. Segundo, a utilização de indicadores padronizados de experiência de cárie permite comparabilidade direta entre os anos de 2010 e 2023, fortalecendo a interpretação temporal das desigualdades. Terceiro, a aplicação de medidas de desigualdade absolutas (SII) e relativas (RII), ajustadas para a variabilidade no número de dentes decíduos, garante maior precisão e validade na mensuração das disparidades sociais em saúde bucal. Quarto, os resultados contribuem para preencher uma lacuna na literatura nacional e internacional sobre a evolução das desigualdades em saúde bucal na infância, fornecendo evidências úteis para subsidiar políticas públicas de equidade em saúde.

Ainda, a interpretação comparativa entre os inquéritos SB Brasil 2010 e 2023 deve ser feita com cautela^13^. Embora ambos compartilhem características metodológicas centrais, como amostragem complexa probabilística por conglomerados, abrangência nacional, realização de exames bucais e entrevistas domiciliares, bem como manutenção de indicadores clínicos semelhantes para a avaliação da cárie dentária, as edições não foram operacionalmente idênticas. Conforme descrito por Vargas et al.^13^, houve mudanças no plano amostral, nos domínios do estudo, no processo de listagem dos domicílios, na composição das equipes de campo e no uso de tecnologias para coleta e monitoramento dos dados, especialmente em 2023. Além disso, possíveis transformações contextuais ocorridas no intervalo entre os levantamentos, incluindo mudanças socioeconômicas, na organização da Atenção Primária à Saúde e no ambiente alimentar infantil, também podem ter influenciado a distribuição da cárie e das desigualdades observadas.

Os achados deste estudo devem ser interpretados à luz do fato de que a renda constitui um marcador relevante das desigualdades sociais em saúde bucal, com associação consistente a piores desfechos bucais, mas que não esgota a complexidade da posição social^4^. Assim, as desigualdades observadas entre os estratos de renda expressam uma dimensão importante da estratificação socioeconômica, sem abranger integralmente outros fatores estruturais e contextuais que também influenciam a ocorrência da cárie na primeira infância. Esse achado indica que os benefícios das ações e políticas voltadas à redução da cárie não têm alcançado de forma equitativa os diferentes grupos sociais, mantendo-se a renda como um determinante central da proteção contra a experiência de cárie em países da América Latina^20^ ^,^ ^21^ ^,^ ^22^ e outros^23^ ^,^ ^24^. Em 2010, crianças oriundas de famílias com maior renda apresentavam cerca de 69% menos risco de desenvolver cárie, proporção que se manteve praticamente estável em 2023, com 66%.

Esse cenário aponta para a necessidade urgente de aprimorar o processo de trabalho na atenção à saúde bucal de crianças, especialmente no que se refere às atividades de prevenção voltadas a crianças em situação de vulnerabilidade socioeconômica. No Brasil, entretanto, ainda existem barreiras importantes, como a ausência da escovação dental supervisionada em muitas escolas públicas, o que representa uma lacuna estratégica no cuidado e poderia ser superada com a ampliação e a qualificação de programas preventivos voltados à primeira infância, como o Programa Saúde na Escola.

Além disso, aspectos culturais são relevantes e, muitas vezes, subestimam a importância da dentição decídua, que frequentemente é vista como dentição transitória e de menor valor clínico para os cuidadores e profissionais, uma percepção que pode prejudicar as estratégias preventivas. Essa percepção, no entanto, ignora o fato de que a presença de cárie em dentes decíduos é um forte preditor da ocorrência futura de cárie em dentes permanentes^25^. Portanto, a atenção adequada à saúde bucal na primeira infância deve ser entendida não apenas como prevenção imediata, mas também como um investimento para reduzir agravos ao longo da vida.

Os determinantes comerciais da saúde têm papel central na persistência e no agravamento da cárie dentária. A disponibilidade, acessibilidade e intensa publicidade de produtos ultraprocessados ricos em açúcares adicionados configuram um dos principais motores da epidemia global de cárie^26^. Achados recentes com dados do SB Brasil 2023 mostram que crianças não brancas, beneficiárias de programas sociais e com uso limitado de serviços odontológicos são mais vulneráveis à cárie, às suas consequências clínicas e à necessidade de tratamento imediato. Esse padrão reforça a necessidade de políticas públicas orientadas pela equidade, com ampliação do acesso à prevenção e ao cuidado odontológico oportuno, especialmente entre grupos socialmente mais vulneráveis^27^.

Além de constatar a ausência de redução significativa nas médias de ceo-d entre os anos 2010 e 2023, observa-se que iniquidades associadas a renda permaneceram inalteradas no período. Tal persistência evidencia que, embora políticas públicas como a expansão da atenção básica e os programas de transferência de renda^28^ tenham contribuído para avanços sociais, não foram suficientes para eliminar as barreiras estruturais que condicionam o risco de cárie nos grupos mais vulneráveis.

A manutenção de valores mais baixos entre crianças de famílias com maior renda, sem redução significativa das disparidades ao longo do período, aponta para a necessidade de políticas mais focalizadas e equitativas, que aliem estratégias universais de prevenção, como fluoretação da água e programas escolares de escovação supervisionada, a ações proporcionais voltadas a contextos de maior vulnerabilidade. Nesse sentido, destacamos a recente revisão de Couto et al.^29^, que sintetiza evidências sobre intervenções com flúor em pré-escolares e reforça que o uso regular de dentifrício fluoretado permanece como a medida preventiva central, sendo estratégias adicionais, como verniz fluoretado, complementares e não substitutivas. A escovação supervisionada potencializa a efetividade do dentifrício fluoretado ao garantir frequência adequada, quantidade correta de pasta e técnica apropriada, além de reduzir desigualdades de acesso ao flúor em contextos socialmente vulneráveis^29^.

Um estudo analisou o efeito da cobertura de programas de transferência de renda na oferta de procedimentos odontológicos na perspectiva do Sistema Único de Saúde (SUS)^28^. Esse estudo concluiu que municípios que ampliaram a cobertura do programa de transferência de renda, que visa à redução de desigualdades, contribuíram para o aumento de restaurações e exodontias, sem produzir efeito significativo na oferta de procedimentos preventivos individuais ou coletivos. Políticas de valorização do salário mínimo, que incluíram correções pela inflação e aumentos reais, têm influência direta sobre o poder de compra das famílias com menor renda, alterando significativamente o rendimento per capita. Essas políticas públicas criam parâmetros naturais para categorizar a renda familiar, refletindo marcos socioeconômicos reconhecidos e objetivos, que foram utilizados no presente estudo^14^ ^,^ ^15^. Cabe lembrar que, entre 2010 e 2023, programas como o Bolsa Família desempenham papel central na ampliação da transferência de renda para famílias em situação de vulnerabilidade, contribuindo para a ascensão social e para a mitigação da pobreza^16^.

 A literatura ainda é limitada e apresenta evidências heterogêneas sobre o efeito de programas de transferência de renda na saúde bucal infantil^30^. Embora o presente estudo não tenha sido desenhado para avaliar diretamente o impacto dessas políticas, nossos achados indicam que as desigualdades de renda permanecem associadas à ocorrência de cárie na primeira infância. Evidências disponíveis sugerem que programas de transferência de renda podem contribuir para reduzir a ocorrência de cárie não tratada em famílias em situação de extrema pobreza^31^; entretanto, tais iniciativas, isoladamente, não são suficientes para eliminar as barreiras estruturais que sustentam o gradiente socioeconômico da doença, reforçando a necessidade de ações intersetoriais articuladas com a organização dos serviços de saúde bucal. Portanto, medidas mais eficazes para reduzir desigualdades precisam ser implementadas, concomitantemente à implementação de estratégias de prevenção mais efetivas. Ainda, futuros estudos podem observar a variação regional destas desigualdades, comparando diferentes regiões do país.

Em conclusão, as desigualdades socioeconômicas em saúde bucal mantiveram-se inalteradas entre 2010 e 2023. Esses resultados sugerem que políticas públicas implementadas no período podem não ter sido suficientes, reforçando a necessidade de estratégias inovadoras e mais equitativas.
